# Spatial distribution and ecological risk assessment of potentially toxic metals in the Sundarbans mangrove soils of Bangladesh

**DOI:** 10.1038/s41598-022-13609-z

**Published:** 2022-06-21

**Authors:** Md Mahfuz Islam, Sayada Momotaz Akther, Md Faruque Hossain, Zakia Parveen

**Affiliations:** 1 Department of Soil, Water and Environment, University of Dhaka, Dhaka, 1000 Bangladesh; 2 OSCM, FBA, American International University-Bangladesh, Dhaka, 1229 Bangladesh; 3 Present Address: Department of Crop and Soil Sciences, North Carolina State University, Raleigh, NC 27695 USA

**Keywords:** Biogeochemistry, Environmental sciences

## Abstract

At present, there are growing concerns over the increasing release of trace metals in the Sundarbans mangrove areas in Bangladesh due to nearby shipbreaking and metallurgical industries, untreated waste discharge, navigation activities, and other natural processes that deposit trace metals into soils. The current study investigated the spatial distribution, contamination level, and ecotoxicity of eight trace metals (Fe, Mn, Cu, Zn, Pb, Cd, Cr, Ni) in Sundarbans soils. Results revealed that all the trace metals except Cr were present in higher concentrations compared to Earth’s shale and/or upper continental crust. Principal component analysis and Pearson correlation showed strong positive correlations (p < 0.05) between Fe, Mn, Cu, and Zn; Ni with Mn and Cr. There were significant associations (p < 0.05) of % clay and total organic carbon (TOC) with Pb-Ni-Cr and negative correlations of pH with all the trace metals. The hierarchical cluster analysis grouped Pb, Ni, and Cd into one distinct cluster, suggesting they are derived from the same sources, possibly from anthropogenic activities. Geo accumulation index (I-geo), enrichment factor (EF), contamination factor (CF), and spatial distribution showed moderately polluted soils with Ni, Pb, and Cd (EF = 3–7.4, CF = 1–2.8, I-geo = 0–0.9) and low pollution by Zn, Cu, Fe, and Mn (EF < 3, CF < 1, I-geo < 0). The ecological risk index (RI) revealed that S-4 (RI = 114.02) and S-5 (RI = 100.04) belonged to moderate risk, and other areas posed a low risk (RI < 95). The individual contribution of Cd (25.9–73.7%), Pb (9.2–29.1%), and Ni (9.6–26.4%) to RI emphasized these metals were the foremost concern in the Sundarbans mangroves due to their long persistence time and high toxicity, even if they were present in low concentrations.

## Introduction

Mangrove forests are important intertidal ecosystems that cover about 1.7 × 10^5^ km^2^ of the shoreline across the tropical and subtropical countries of the world^1,2^. These forests support various ecological services, such as coastal protection, carbon sequestration, fishery, and habitats for diverse fauna and flora^3,4^. Despite their importance, mangroves are endangered ecosystems, declining globally at a 1–2% rate per year due to pollution from anthropogenic pressures^5^. Mangrove soils are considered an essential sink for pollutants because of their extensive capacity to retain various organic and inorganic contaminants^6,7^. Among the contaminants, trace metals are particularly concerning due to their non-biodegradability, long persistence, high toxicity and potential to accumulate in the tissues of inhabiting organisms such as fishes, plants, and birds^8–10^. Furthermore, trace metals may not be constantly fixed in soil and sediments; instead, they may be recycled into the marine estuarine environment causing mangroves to act as sources of pollution^11,12^. For instance, plants growing in contaminated soils absorb toxic metals and accumulate them in their biomass. The consumption of these plants by mangrove inhibiting animals results in the transfer of toxic metals in the food web^13^.

Although trace metals are naturally occurring in soil by weathering of parent rocks, soil contamination rates due to anthropogenic activities largely outplace natural processes. The world's largest mangrove, Sundarbans, is undergoing environmental degradation due to rapid agricultural and aquacultural activities, intensive fishing, expanding human settlements, tourism, industrial effluents, and oil spills^14–16^. Khulna Shipyard discharges substantial amounts of solid, liquid, and gaseous pollutants such as oil, asbestos, persistent organic compound, trace metals especially, iron, lead, nickel, cadmium, zinc, copper, chromium, manganese, etc. into the Posur River that are hazardous to Sundarbans environment^17,18^. In addition, several steel industries, Mongla Port, Goalpara Power Plant, Khulna Newsprint Mill and hardboard mills in the Khalishpur Industrial Belt also dispose of their untreated wastes in the upstream Rupsa and Bhairab rivers^18–20^. This represents an environmental crisis, which may be further exacerbated by coal combustion and navigation activities due to the operation of the Rampaul Power Plant that is located only 14 km away from Sundarbans mangrove boundary. Hence, it is necessary to evaluate the distribution of potentially toxic metals in soils to understand the present pollution level and ecological risk in Sundarbans.

Over the years, some research has been conducted regarding trace metals pollution on the Indian Sundarbans^16,21–23^_._ In contrast, only a limited number of studies have been performed on the Bangladesh Sundarbans and the ones that attempted to do so did not evaluate the environmental risk of potentially toxic metals^19,20^. Moreover, these studies were based on only a limited number of samples (~ 10) which may not adequately represent the spatial viability of mangrove areas in the Bangladesh Sundarbans. The current study is the first work conducted to examine the environmental risk of trace metals contamination covering all the four administrative ranges (Chandpai, Sharankhola, Nalian, and Buri Goalini) of the Bangladesh Sundarbans. In addition, The ArcGIS-based spatial distribution of trace metals will provide their contamination patterns even in unsampled spots of this area. Such a study would be helpful to compile baseline data for future monitoring and conservation in the Sundarbans mangrove forest and areas of similar types.

Recently, several soil trace metals remediation methods (physical, chemical, and biological) were implemented to mitigate trace metals contamination issues^24^. Physical remediation techniques are typically used for small-scale treatments and are not economically viable forest soil remediation options^25^. Chemical leaching and fixation are not permanent solutions since mangrove plants are deep-rooted and metals may further get released into the soil under conducive conditions^25–27^. Microbial and phytoremediation have received significant attention nowadays to remediate trace metals from contaminated sites. Difficulty in phytoremediation and microbial transformation is to select a particular species for a particular type of metals remediation^24,25^. As a result, evaluating the levels of trace metals contamination and associated ecological risk is important to determine which remediation method is better suited for the conditions of the Sundarbans ecosystem. Numerous soil pollution indices are currently being used worldwide to evaluate the toxic metals pollution in soil and sediment. Among the various risk assessment techniques reported in the literature, enrichment factor (EF), geo-accumulation index (I-geo), contamination factor (CF), and potential ecological risk index (RI) were used to estimate the trace metals contamination in the Sundarbans mangrove soils in Bangladesh^1,28,29^. The enrichment factor and geo accumulation index, proposed by Muller^30^ and Taylor^31^, respectively, are used to recognize the probable human-induced influences. The I-geo and EF are determined from trace metals concentration and their soil geochemical background values. Hakanson^32^ suggested CF and RI to quantify the level of heavy metals contamination in soils and sediments and the overall ecological risk of multiple trace metals.

Keeping in mind the potential ecological catastrophe of trace metals, the primary aims of this study are to (i) evaluate distributions of eight trace metals in Bangladesh Sundarbans soils and (ii) assess the level of contamination and potential ecological risks using the environmental indices mentioned above.

## Materials and methods

### Study area

Sundarbans mangrove forest is formed by the confluence of mighty Ganges, Brahmaputra, Meghna, and Padma rivers, located in the vast delta of the Bay of Bengal, Southern Bangladesh. This forest encompasses an area of 10,029 km^2^, shared between Bangladesh (6017 km^2^) and West Bengal, India (about 4012 km^2^)^33^. The home of the Royal Bengal Tiger is a tidal depositional ecosystem consisting of a complex network of islands and estuaries dissected by numerous rivers, creeks, and channels. The tropical southwest monsoon controls river water discharge, and ∼ 95% of the sediment load is transported to the coast of the Bay of Bengal from May to September due to higher rainfall^34–36^. Several studies have estimated that the Ganges river carries a load of about 485 to 1600 million tons of suspended sediment per year^37^. The climate of the Sundarbans mangrove area is humid sub-tropical and is relatively consistent with the non-mangrove adjacent regions. The average annual temperature, rainfall, and humidity of Sundarbans mangrove forest vary from 17–32 °C, 1640–2000 mm, and 70–80%, respectively. Generally, high temperature is observed from mid-March to mid-June, and minimum temperature is observed in December and January. Rainfall increases from the west to east side of Sundarbans, and about 80% is from May to October. The highest and lowest humidity prevail in June–October and February, respectively^38^.

This pristine mangrove has been undergoing environmental degradation over the years by anthropogenic activities, for example, shipbreaking industries, iron and steel mills, port, fishing, tourism, aquaculture and so on^33^. The biogeochemical process and biodiversity of the Sundarbans have been seriously affected by effluent discharge from industries, changes in land-use patterns, oil spills from navigation, runoff from agricultural fields, etc.

### Sample collection and preparation

**Figure 1:**
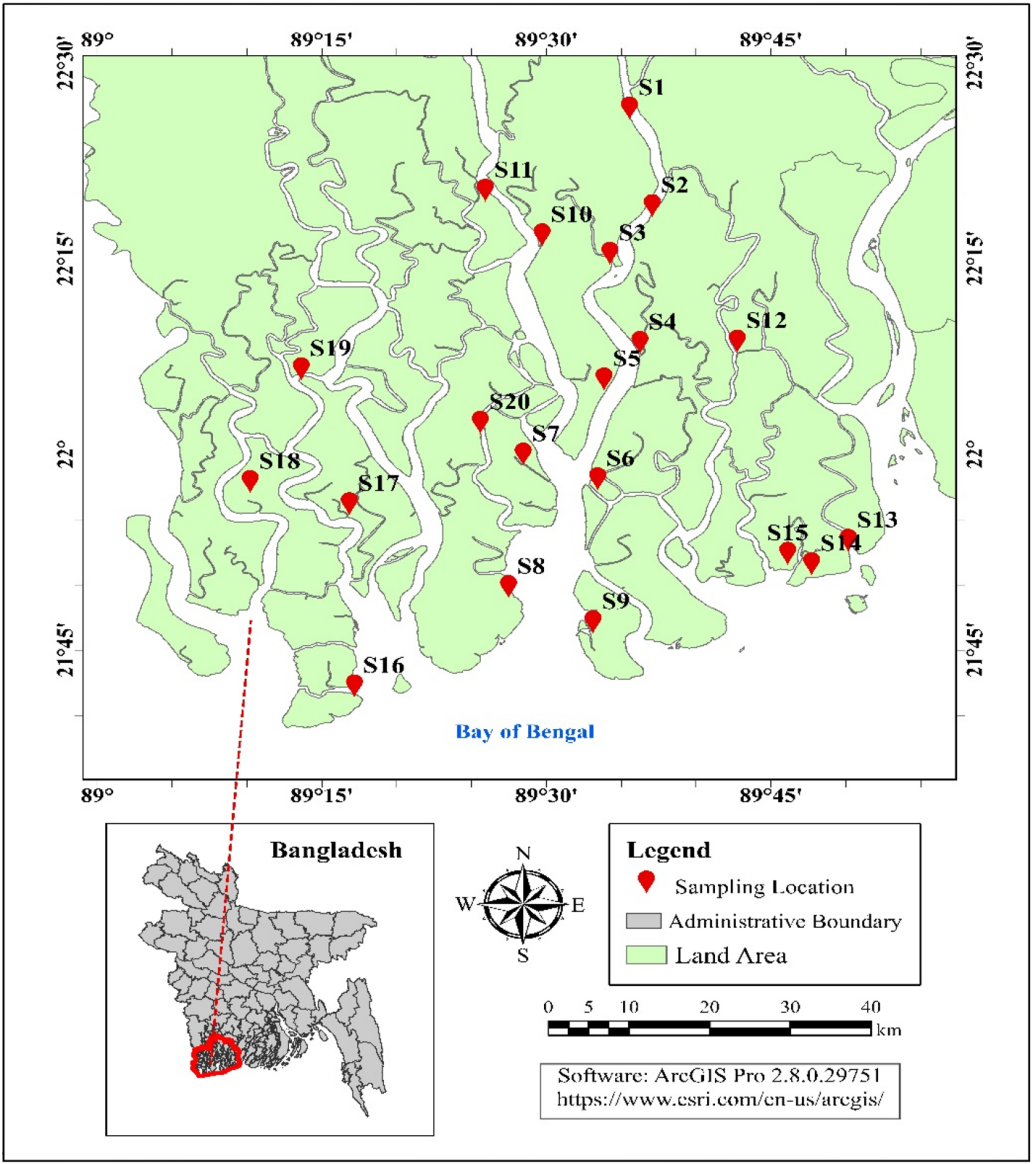
Study area map of the Sundarbans mangrove forest.

Twenty locations were selected in the Sundarbans mangrove according to varying anthropogenic pressures and the feasibility of sampling. The sampling area covered all the four administrative ranges viz Chandpai, Sharankhola, Nalian, and Buri Goalini of the Bangladesh Sundarbans mangrove forest (Fig. 1). The bulk of composite soils from 0 to 15 cm depth were collected using a soil auger and put into separate zipped lock polyethylene bags, labeled, and brought into the laboratory for further analyses. Approximately 10 × 10 m^2^ area was selected in each position, and one composite sample was taken from five sub-samples (4 corners and center). Collected soil samples were air-dried for 2 weeks at the laboratory, and visible roots, leaves, and other debris were removed. The resulting dried soil samples were gently broken down by a wooden hammer. The crushed soils were screened to pass through 2 mm (physical analyses) and 0.5 mm (chemical analyses) stainless steel sieves, mixed thoroughly, and preserved in clean plastic containers.

### Analytical method

A microwave digestion system (MARSXpress, CEM GmbH, Kamp-Lintfort, Germany) and Atomic Absorption Spectrophotometer (VARIAN AA240, New Jersey, USA) were used to extract and analyze the total concentrations of the eight examined trace metals. For this determination, 0.5 g soil was digested in a Teflon vessel with 26 mL of HNO_3_, HCl, HF in a ratio of 9:3:1 at 105 °C for 2 h^1^. After dissolution, extracts were filtered via Whatman-42 filter paper into 10% HNO_3_ acid rinsed dry volumetric flask, volumed up to the mark using Milli Q water and kept in the refrigerator at 4 °C. The pH (Soil: Deionized water = 10: 25, w/v) of samples were recorded electrochemically using a calibrated HACH pH meter. Soil textures were determined by the hydrometer method as described by Bouyoucos^39^. Finally, the Wet Oxidation method of Walkley and Black^40^ was used to measure total organic carbon (TOC) content.

### Quality control and quality assurance

Merck Germany supplied analytical grade acids and reagents were used in this study. The solutions were prepared with Milli Q water (18.2 MΩ/cm Millipore Milli Q Plus system; MA, USA). The experimental apparatuses were dipped into 10% HNO_3_ acid overnight, rinsed thoroughly with Milli Q water, and dried before use. Quality assurance and quality control were assessed using triplicates, method blanks, reagent blank, and certified reference materials (CRM). Triplicates analyses were done for each soil sample, and method and reagent blanks were run simultaneously for all the trace elements. Five replicate blank samples were digested to determine the detection limit (LOD) and found < 0.008 mg kg^−1^ for all the metals except Fe (Table 1). The accuracy of the analytical procedure of AAS was checked by the analysis of certified reference materials (CRM) SRM 2709a from San Joaquin Valley Soil, California, USA (Table 1). Calibration curves with an R^2^ value of a minimum of 0.9985 were considered for concentration calculation. Limit of Detection (LOD) was determined by using LOD = Reagent blank + 3 s formula, where s is the standard deviation. Reagent blank was calculated from seven blank replicates, and standard deviation (s) was calculated from seven replicates of SRM 2709a soil samples.

**Table 1:** Detection limit (LOD) and % recovery of the studied eight trace metals.

Trace metal	Wavelength (nm)	LOD (mg kg^−1^)	NIST- SRM 2709a		
			Certified value (mg kg^−1^)	Measured value (mg kg^−1^)	% Recovery
Fe	248.3	0.063	3.36 ± 0.07%	3.32 ± 0.09%	95.24
Mn	279.5	0.0081	529.00 ± 18.00	516.54 ± 15.60	97.73
Cu	324.8	0.0061	33.90 ± 0.50	33.10 ± 0.80	97.64
Zn	213.9	0.0086	103.00 ± 4.00	101.12 ± 2.57	98.17
Pb	217.0	0.005	17.30 ± 0.10	16.81 ± 0.16	97.11
Cr	357.9	0.0087	130.00 ± 9.00	127.85 ± 4.92	98.35
Ni	232.0	0.0035	85.00 ± 2.00	82.37 ± 2.43	96.91
Cd	228.8	0.0086	0.371 ± 0.002	0.354 ± 0.005	95.42

### Statistical analyses

Experimental data analyses were carried out using Minitab-18 and Microsoft Office-10 software. Pearson correlation was run to reveal the relationship between trace metals and selected soil properties. In addition, the associations between sampling sites and between trace metals load were examined by factor analysis (Principal Component Analysis and Hierarchical Cluster Analysis) in Minitab-18 software. Relationships were assumed as significant at p < 0.05.

### Risk assessment

The utmost importance of assessing a site and determining how to cope with soil management is quantifying the ecological risk associated with soil pollution. In this study, four soil contamination parameters were used to evaluate the level of soil contamination and ecological risks in the Sundarbans mangrove (Table 2).

**Table 2:** Description of the soil contamination indices.

Environmental parameter	Equation	Soil quality	References
Geo-accumulation index (I-geo)	I-geo = ${\mathrm{log}}_{2}\left(\frac{Cn}{1.5 Bn}\right)$… (i) Herein, Cn and Bn are the measured concentration and geochemical background values of the element n in the Earth’s crust. The factor of 1.5 is due to lithogenic effects	Igeo ≤ 0 practically unpolluted; 0 < Igeo ≤ 1; unpolluted to moderately polluted; 1 < Igeo ≤ 2 moderately polluted; 2 < Igeo ≤ 3 moderately to strongly polluted; 3 < Igeo ≤ 4 strongly polluted; 4 < Igeo ≤ 5 strongly to extremely polluted; and Igeo > 5 extremely polluted	^30^
Enrichment factor (EF)	EF = $\frac{\left[\left(Mc/Mr\right)\right]S}{\left[\left(Mc/Mr\right)\right]b}$….. (ii) wherein, Mc = studied metal conc., Mr = reference material conc., s = sample, b = background	EF < 1 means no enrichment; 1 < EF < 3 means minor enrichment; 3 < EF < 5 indicates moderate enrichment; 5 < EF < 10 indicates moderately severe enrichment; 10 < EF < 25 is severe enrichment; 25 < EF < 50 means very severe enrichment; and EF > 50 is extremely severe enrichment	^31,41^
Contamination factor (CF)	$\mathrm{CF}=\frac{\mathrm{Mc}}{\mathrm{Bc}}$……… (iii) Mc and Bc are measured and background concentrations of the metal	CF < 1: low contamination; 1 ≤ CF < 3: moderate contamination; 3 ≤ CF < 6: considerable contamination; CF > 6: very high contamination	^32^
Potential ecological risk index (RI)	E = T × CF………… (v) RI = $\sum E$…………. (vi) where, E = factor of RI T = biological toxicity factor	RI < 95 implies low potential ecological risk; 95 < RI < 190 implies moderate ecological risk; 190 < RI < 380 means considerable ecological risk; RI > 380 indicates very high ecological risk	^32^

Since background concentrations of metals were not available for the Sundarbans area, earth surface rock standard concentrations^42^ were used as background values throughout the study because sedimentary rocks extensively cover this area. The background value of Cu had been taken from Turekian and Wedepohl^43^. Typically, elements like Al, Mn, Fe, etc. are employed as reference material in enrichment factor analysis^44^. We selected Fe as the reference one in this experiment because ~ 98% of Fe comes from natural sources^45^. The biological toxicity response factor in determining the potential ecological risk index of the selected metals are as follows: Zn and Mn are 1; Cr is 2; Ni, Cu & Pb are 6, Cd is 30^32,46^.

### Consent to participate

The article does not contain any studies with human participants or animals performed by any of the authors.

### Consent to publish

We declare that this manuscript is original, has not been published before, and is not being currently considered for publication elsewhere.

## Results and discussions

### Soil characteristics

Soil texture, percentage total organic carbon (% TOC) and soil pH are illustrated in Table 3. Organic carbon of the twenty study sites ranged from 1.26 ± 0.08 to 8.06 ± 0.32% with a mean value of 2.31 ± 1.80%. Organic carbon in 18 out of 20 samples was below 3% in the Sundarbans soils which were very low compared to global mean carbon content (7.9%) for a mangrove forest in tropical region^35^. The Bangladesh Sundarbans contained lower carbon because local organic matters are exported to the coastal zones by tidal activities or poor adsorption of organics to finer soil particles^14,47,48^. The soil pH of the studied areas was slight to moderately alkaline except in S-1, S-2 and S-9. The higher soil acidity implied anthropogenic inputs that may alter the soil physical and chemical properties and raise the pH ~ 1.5 units than native soils^22^. The soils were medium to well-sorted, ranging in texture between loam to silt, and followed the order of silt (≥ 49.70%) > clay (≥ 5.50%) > sand (≥ 2.94%). Silt and clay were dominant and probably responsible for retaining trace metals in Sundarbans environments. The grain size distribution showed a decreasing trend of sand and increasing silt contents from S-1 to S-9 possibly because these sampling sites are located from the upper to lower stream of the Posur River. The higher sand percentage at S-1, S-11, and S-12 indicate their positions in active depositional banks of river meander, whereas S-15 and S-16 attributed the dominance of marine inputs via tidal channels. The irregular distribution of clay in all the soils and sand-silt in the rest of the soils suggested vigorous estuarial mixing, suspension, resuspension, flocculation, deflocculation processes^23^.

**Table 3:** Soil physical and chemical properties of the Sundarbans mangrove.

Sample ID	Latitude	Longitude	% sand	% silt	% clay	Textural class	% TOC	pH
S-1	22.427500° N	89.592500° E	10.22	77.04	12.74	Silt loam	2.96 ± 0.05	6.95 ± 0.05
S-2	22.302777° N	89.617777° E	7.57	82.78	9.65	Silt	1.89 ± 0.03	6.91 ± 0.01
S-3	22.241888° N	89.570666° E	6.33	84.04	9.63	Silt	1.67 ± 0.03	7.09 ± 0.01
S-4	22.128020° N	89.604166° E	5.51	78.39	16.10	Silt loam	2.77 ± 0.02	7.44 ± 0.01
S-5	22.081331° N	89.564176° E	5.60	85.04	9.36	Silt	1.44 ± 0.07	7.21 ± 0.01
S-6	21.953751° N	89.557123° E	4.38	83.82	11.80	Silt loam	1.64 ± 0.03	7.31 ± 0.01
S-7	21.985448° N	89.474192° E	4.17	90.33	5.50	Silt	1.26 ± 0.08	7.44 ± 0.01
S-8	21.816111° N	89.457777° E	4.38	81.39	14.23	Silt	2.40 ± 0.03	7.12 ± 0.01
S-9	21.771017° N	89.551863° E	3.15	77.19	19.66	Silt loam	8.06 ± 0.02	6.12 ± 0.02
S-10	22.265424° N	89.495259° E	6.25	85.15	8.60	Silt	1.79 ± 0.05	7.40 ± 0.03
S-11	22.322373° N	89.431939° E	16.80	76.50	6.70	Silt loam	1.29 ± 0.06	7.42 ± 0.01
S-12	22.129722° N	89.712500° E	25.80	49.70	24.50	Loam	6.63 ± 0.19	7.39 ± 0.03
S-13	21.875100° N	89.836111° E	2.94	82.66	14.40	Silt loam	1.95 ± 0.02	7.90 ± 0.02
S-14	21.845278° N	89.795277° E	4.05	89.26	6.69	Silt	1.47 ± 0.03	7.95 ± 0.01
S-15	21.858611° N	89.768611° E	21.60	68.60	9.80	Silt loam	1.36 ± 0.32	8.09 ± 0.01
S-16	21.689235° N	89.286209° E	13.30	78.90	7.80	Silt loam	1.52 ± 0.06	8.02 ± 0.06
S-17	21.921881° N	89.280537° E	9.72	82.98	7.30	Silt	1.68 ± 0.22	7.55 ± 0.10
S-18	21.950176° N	89.170176° E	4.18	89.32	6.50	Silt	1.40 ± 0.02	7.44 ± 0.03
S-19	22.094297° N	89.227010° E	4.28	89.05	6.67	Silt	1.60 ± 0.02	7.64 ± 0.01
S-20	22.025855° N	89.426393° E	3.56	88.04	8.40	Silt	1.48 ± 0.04	7.32 ± 0.01
*Min*	–	–	2.94	49.70	5.50	–	1.26 ± 0.08	6.12 ± 0.02
*Max*	–	–	25.80	90.33	24.50	–	8.06 ± 0.32	8.09 ± 0.01
*Mean*	–	–	8.19	81.01	10.80	–	2.31	7.39
*SD*	–	–	6.43	9.16	4.91	–	1.80	0.45

*Min.* minimum, *Max* maximum, *SD* standard deviation.

### Distribution of potentially toxic metals

Concentrations of total trace elements in the Sundarbans soils are summarized in Table 4 and the concentrations in this study ranged from 20,920 ± 804.1 to 38,432.5 ± 172.5 μg g^−1^ for Fe, 469.76 ± 12.5 to 803.14 ± 50.6 μg g^−1^ for Mn, 24.38 ± 0.23 to 41.83 ± 0.08 μg g^−1^ for Cu, 46.05 ± 3.30 to 72.07 ± 3.31 μg g^−1^ for Zn, 16.57 ± 1.20 to 39.60 ± 4.53 μg g^−1^ for Pb, 0.07 ± 0.03 to 0.56 ± 0.02 for Cd, 47.65 ± 4.70 to 103.95 ± 22.25 μg g^−1^ for Ni and 15.15 ± 0.04 to 48.84 ± 0.64 μg g^−1^ for Cr. The highest concentration of soil Zn, Pb, Ni, and Cr belonged to the S-9 soils. In contrast, the maximum Fe-Cu, Mn and Cd concentrations were determined in S-1, S-8, and S-4, respectively. Moreover, all the studied metals were higher in the Posur river adjacent areas (S-1 to S-9) compared to S-10 to S-20 soils. If Fe is excluded, Pb and Ni had higher standard deviations, implying less uniform distribution over the sampling area and human activities influence these metals. Nearby upstream Mongla port, Khulna Shipbreaking Industry, iron-steel production, electroplating activities and intensive navigation activities in the Posur, Rupsa and Bhairab rivers might be possible reasons for this variation. Based on the average values, trace metal concentrations in the Bangladesh Sundarbans mostly followed the sequence: Fe > Mn > Ni > Zn > Cr > Cu > Pb > Cd. Meanwhile, Kumar and Ramanathan^47^ and Banerjee et al.^16^ reported a higher Zn distribution than Ni in Indian Sundarbans. Many researchers reported higher Zn concentration^28,49,50^**,** some found higher Pb^51,52^, while Chen et al.^53^ found Cr as the most abundant metal in soil. The Studied metal concentrations are compared with the concentrations in earth’s shale by Turekian and Wedepohl^43^, upper continental crust (UCC) by Taylor and McLennan^54^ (Table 4). Four metals, mainly zinc, copper, iron, and manganese concentrations were lower than the Earth’s shale value but higher than the UCC. On the contrary, lead, cadmium, and nickel concentrations were elevated than that of both shale and UCC values.

**Table 4:** Distribution of the trace metal concentrations (µg g^-1^) in the Sundarbans mangrove soils.

Sample	Fe	Mn	Cu	Zn	Pb	Cd	Ni	Cr
S-1	38,432.5 ± 172.5	628.25 ± 11.25	41.83 ± 0.08	55.98 ± 0.11	32.67 ± 5.41	0.31 ± 0.01	70.44 ± 2.13	45.75 ± 0.42
S-2	36,137.5 ± 232.5	713.25 ± 26.255	29.37 ± 0.95	63.37 ± 0.28	25.20 ± 3.28	0.21 ± 0.01	101.79 ± 3.04	33.80 ± 0.49
S-3	35,570.0 ± 290.0	702.15 ± 5.0	39.88 ± 0.63	59.89 ± 2.83	22.54 ± 1.65	0.18 ± 0.02	75.63 ± 2.14	41.30 ± 0.42
S-4	31,787.5 ± 837.9	653.20 ± 11.18	29.00 ± 1.50	47.83 ± 2.53	32.4 ± 4.15	0.56 ± 0.02	92.61 ± 5.11	34.90 ± 0.35
S-5	35,552.5 ± 121.0	665.80 ± 22.50	37.48 ± 0.70	58.60 ± 0.78	24.44 ± 3.77	0.49 ± 0.03	78.33 ± 4.42	40.40 ± 0.28
S-6	32,762.5 ± 201.9	666.15 ± 15.0	35.94 ± 0.77	59.90 ± 0.98	19.36 ± 2.66	0.34 ± 0.01	74.85 ± 11.26	36.60 ± 0.84
S-7	33,135.0 ± 641.1	636.60 ± 27.3	31.10 ± 0.23	52.34 ± 0.36	18.00 ± 1.56	0.17 ± 0.03	69.15 ± 9.32	34.00 ± 0.59
S-8	37,932.5 ± 257.5	803.14 ± 50.6	38.29 ± 10.0	59.10 ± 2.52	27.9 ± 2.40	0.23 ± 0.01	85.60 ± 3.21	41.95 ± 0.60
S-9	32,760.7 ± 655.0	665.1 ± 35.0	37.17 ± 0.88	72.07 ± 3.31	39.60 ± 4.53	0.11 ± 0.01	103.95 ± 22.25	48.84 ± 0.64
S-10	29,592.5 ± 350.3	559. 7 ± 23.67	36.17 ± 0.28	63.52 ± 0.85	19.17 ± 1.01	0.08 ± 0.01	75.35 ± 4.64	32.50 ± 0.46
S-11	31,365.0 ± 575.5	581.84 ± 17.50	24.38 ± 0.23	53.27 ± 2.46	28.01 ± 2.10	0.1 ± 0.02	60.82 ± 2.09	35.65 ± 0.32
S-12	28,190.0 ± 525.4	735.70 ± 42.5	33.92 ± 0.33	62.45 ± 0.35	30.67 ± 1.67	0.10 ± 0.03	87.00 ± 2.90	40.00 ± 0.35
S-13	26,105.1 ± 440.0	574.84 ± 32.5	30.58 ± 0.58	58.52 ± 0.16	21.74 ± 0.63	0.25 ± 0.03	84.60 ± 3.27	42.75 ± 0.32
S-14	27,192.5 ± 297.5	539.48 ± 22.3	25.75 ± 0.25	47.97 ± 0.54	29.79 ± 6.03	0.09 ± 0.01	67.65 ± 4.09	24.61 ± 0.71
S-15	30,902.5 ± 912.5	469.76 ± 12.5	28.82 ± 0.08	48.29 ± 1.90	17.14 ± 0.63	0.07 ± 0.02	62.23 ± 4.21	15.15 ± 0.04
S-16	20,920.0 ± 804.1	478.24 ± 82.5	27.05 ± 0.50	46.05 ± 3.30	32.81 ± 3.31	0.31 ± 0.04	71.25 ± 5.00	24.22 ± 0.92
S-17	26,125.3 ± 144.2	619.36 ± 62.1	32.27 ± 0.18	61.21 ± 1.19	33.35 ± 2.52	0.14 ± 0.01	60.55 ± 3.73	34.55 ± 0.21
S-18	28,310.3 ± 318.6	637.56 ± 47.5	31.51 ± 0.05	63.28 ± 1.19	19.50 ± 0.85	0.11 ± 0.03	55.80 ± 3.84	35.23 ± 0.68
S-19	33,960.0 ± 1656.8	593.9 ± 10.0	24.87 ± 0.48	71.76 ± 8.50	25.00 ± 1.57	0.09 ± 0.01	47.65 ± 4.70	22.35 ± 0.78
S-20	22,207.5 ± 365.4	472.78 ± 19.0	27.70 ± 0.40	51.13 ± 2.15	16.57 ± 1.20	0.09 ± 0.01	53.35 ± 4.02	20.83 ± 0.57
*Min*	20,920.0 ± 804.1	469.76 ± 12.5	24.38 ± 0.23	46.05 ± 3.30	16.57 ± 1.20	0.07 ± 0.03	47.65 ± 4.70	15.15 ± 0.04
*Max*	38,432.5 ± 172.5	803.14 ± 50.6	41.83 ± 0.08	72.07 ± 3.31	39.60 ± 4.53	0.56 ± 0.02	103.95 ± 22.25	48.84 ± 0.64
*Mean*	30,947.07	623.01	32.15	57.83	25.79	0.202	73.93	34.27
*SD*	4865.478	89.82	5.19	7.47	6.54	0.14	15.36	8.85
Shale	47,200	850	45	95	NA	0.3	68	90
UCC	35,000	600	25	71	16	0.098	50	85

*Min*. minimum, *Max*. maximum, *SD* standard deviation, UCC upper continental crust.

The lower concentration of Cr in all the studied locations indicated that Cr is not actively releasing from the nearby anthropogenic sources. The concentrations of Fe, Mn, Cu, Zn, Pb, Cd, and Ni in most soil samples exceeded the geochemical background values, indicating that contamination is present in Sundarbans soils, and investigation of environmental risks is required.

Trace metals concentrations in worldwide mangrove soils were surveyed in literature and summarized in Table 5. Iron concentration was higher than that of mangroves in India (1409.67 µg g^−1^) by Kader and Sinha^22^, Panama (9827 µg g^−1^) by Guzmán and Jiménez^55^ and Colombia (15,593 µg g^−1^) by Perdomo et al.^56^ but lower than Roy et al.^21^. Compared with the other studies globally, Ni concentrations in Bangladesh Sundarbans were also higher than the described values in India, Brazil, China, Malaysia, Saudi Arabia, Panama, and Colombia (Table 5). Contrary, Cd was lower and other metals were similar to worldwide mangroves. These spatial variations of potentially toxic elements in Bangladesh Sundarbans and worldwide mangroves might be ascribed to the difference in point-nonpoint sources and loads, hydrodynamics, tidal settings and so on^27^ due to churning, bioturbation, flocculation-deflocculation, and suspension^57^.

**Table 5:** Comparison of trace metals concentrations (µg g^−1^) in worldwide mangrove soils.

Location	Fe	Mn	Cu	Zn	Pb	Cd	Ni	Cr	References
Sundarbans, Bangladesh	38,432.5 ± 172.5	803.14 ± 50.6	41.83 ± 0.08	72.07 ± 3.31	39.60 ± 1.53	0.56 ± 0.02	103.95 ± 20.25	48.84 ± 0.64	This study
Sundarbans, India	1409.67	978.41	164.64	–	18.54	1.77	–	–	^22^
Sundarbans, India	46,867	–	36.76	97.97	52.9	1.98	50.98	491.73	^21^
China	–	–	77.59 ± 4.64	282.09 ± 19	63.82 ± 3.9	3.47 ± 0.27	40.19 ± 3.54	95.43 ± 6.86	^1^
Punta Mala Bay, Panama	9,827	295	56.30	105.00	78.20	< 10	27.30	23.30	^55^
Cienaga Grande, Colombia	15,593	623	23.30	91.00	12.60	1.92	32.50	13.20	^56^
Brazil	–	273	80	610	130	–	12	–	^58^
Matang, Malaysia	–	–	40.54	80.08	8.14	1.35	17.34	–	^59^
Arabian Gulf, Saudi Arabia	–	–	67.09	64.28	40.54	1.09–7.30	32.00	77.18	^60^

### Spatial distribution

**Figure 2:**
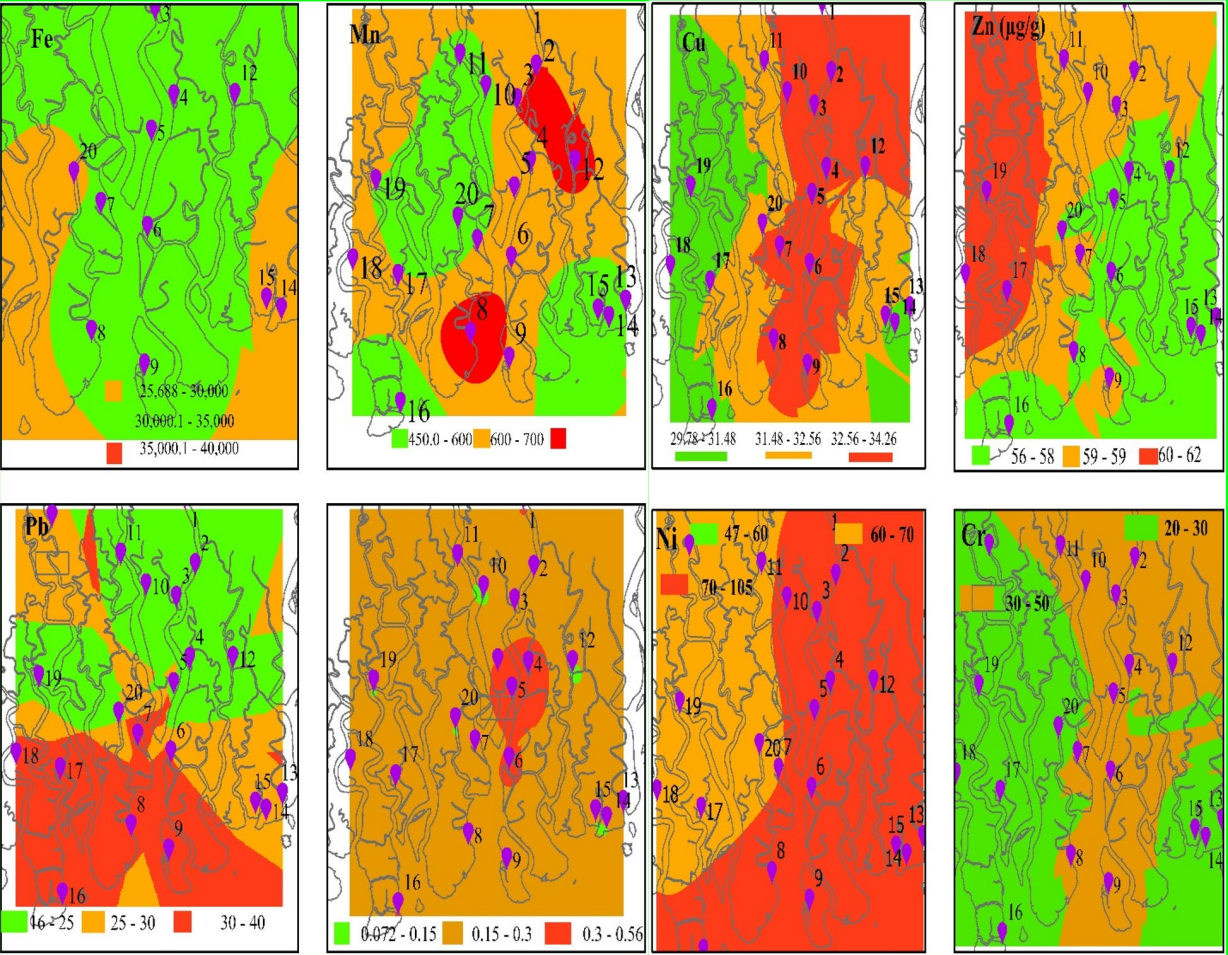
Spatial distribution of trace metals in Bangladesh Sundarban Soils (ArcGIS Pro).

The spatial distribution of trace metals in the Bangladesh Sundarban mangrove was obtained by ordinary Kriging interpolation in ArcGIS Pro 2.8.0.29751, licensed under North Carolina State University (https://www.esri.com/en-us/arcgis/products/arcgis-pro/overview). Spatial interpolation enables us to evaluate trace metals concentrations even in the unsampled areas. Spatial distributions of the eight studied metals are presented in Fig. 2.

The spatial distribution map is also a precious tool to identify hotspots of trace metals pollution and delineate the safe and unsafe spots in a study area. Cu, Cd, and Ni showed similar spatial distribution patterns, whereas other patterns were varied. The trace metals polluted hotspots in the Sundarbans mangrove are located in the nearby areas of the Rupsa and Bhairab rivers. This clearly indicates human activities in this area that posed environmental risks and will require further vigilance. Industrial developments in upstream of the Sundarbans could be the main reasons for the high concentrations of Pb, Cd, and Ni.

### Multivariate analysis

#### Pearson correlation study

Before multivariate analysis, Pearson correlation coefficient was performed to strengthen the relationships between the trace metals within the samples and with soil properties (Table 6). Correlation analysis is an excellent tool for finding information about similar pathways or origins of environmental contaminants^28^. Egbueri et al.^49^ divided correlation coefficients into strong (r > 0.7), moderate (0.5 < r < 0.7) and week (r < 0.5) correlations. A strong to moderate correlation of Fe, Mn, Cu, and Cr indicated their origin from similar sources and its possibly geogenic mostly and a minor of anthropogenic activities. In all the studied soils, the interrelationship of trace metals showed no significant relationship of Cd and Pb with any of the metals and Ni with only Mn and Cr due to their specific human-induced activities. The positive correlations of Fe and/or Mn with Cu, Zn, and Cr indicated that precipitation of these elements with Fe–Mn oxides and hydroxides play a significant role in mangrove systems^61–63^. The soil pH negatively affected trace metals distribution in Sundarbans soils and supported the inverse interaction between trace metals and soil pH. A significant positive correlation of total organic carbon (TOC) with Zn, Pb, Ni and Cr implied that organic carbon acts as a host for these metals in the Sundarbans mangrove soils.

**Table 6:** Pearson correlation coefficient of trace metals and soil properties in Sundarbans soils.

Metals	Fe	Mn	Cu	Zn	Pb	Cd	Ni	Cr	% Clay	%TOC
Mn	0.673**									
Cu	0.541**	0.573**								
Zn	0.356	0.493*	0.349							
Pb	0.055	0.253	0.111	0.122						
Cd	0.261	0.223	0.247	-0.298	0.179					
Ni	0.323	0.560**	0.394	0.193	0.422	0.366				
Cr	0.477*	0.726**	0.72**	0.442*	0.410	0.285	0.601**			
% Clay	0.131	0.473*	0.399	0.252	0.449*	0.182	0.684**	0.533**		
TOC	0.001	0.293	0.218	0.438*	0.549**	− 0.248	0.522*	0.431*	0.855**	
pH	− 0.548**	− 0.597**	− 0.599**	− 0.576**	− 0.347	− 0.053	− 0.539**	− 0.685**	− 0.402	− 0.60**

*Indicates significance at 0.05 probability level and **indicates significance at 0.01 probability level.

#### Principal component analysis

**Figure 3:**
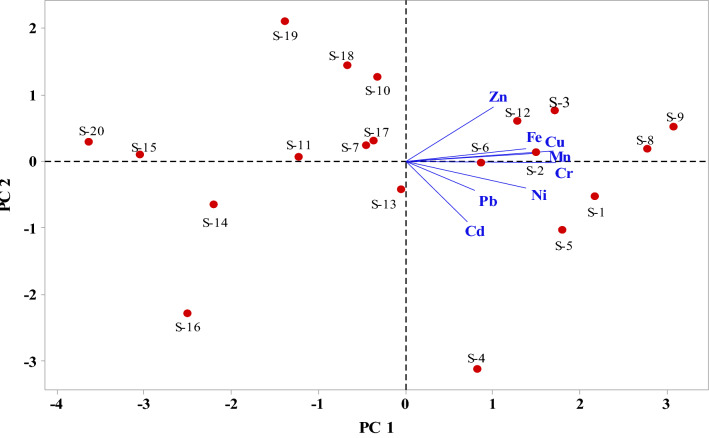
Score plot of studied locations and loading plot of studied trace metals for PC 1 and PC 2.

Principal component analysis (PCA) transformed the original measurement variables into an uncorrelated linear combination of variables to assess the relationship between studied trace metals and the sampling locations (Fig. 3). The PCA analysis yielded three significant PCs (PC 1, PC 2 and PC 3) and accounted for 77.6% of the total variances among eight variables. The significant PCs were selected based on Kaiser criterion (eigenvalue > 1)^64^.

The PC 1 explained 47.0% of the calculated variance, showing high positive loadings for Pb, Cd and Ni and low positive loadings for Fe, Mn, Cu, Cr, and Zn. Three samples (S-1, S-4, S-5) are characterized by high values for Pb, Cd and Ni, while six samples (S-2, S-3, S-6, S-8, S-9, S-12) showed high values for Fe, Mn, Cu, Cr, and Zn in PC 1. Nearby metallurgical industries, shipbreaking industries, oils spill from navigations might be the dominating source for high loadings of Pb, Cd, Ni in PC 1**.** In PC 2 and PC 2, no significant loadings of any variable were found, responsible for 17.4% and 13.2%, respectively of the total measured variances. The positive inter-elemental relationships demonstrated that long-term anthropogenic activities probably drive these trace metals above the background values^48^.

#### Hierarchical cluster analysis

**Figure 4:**
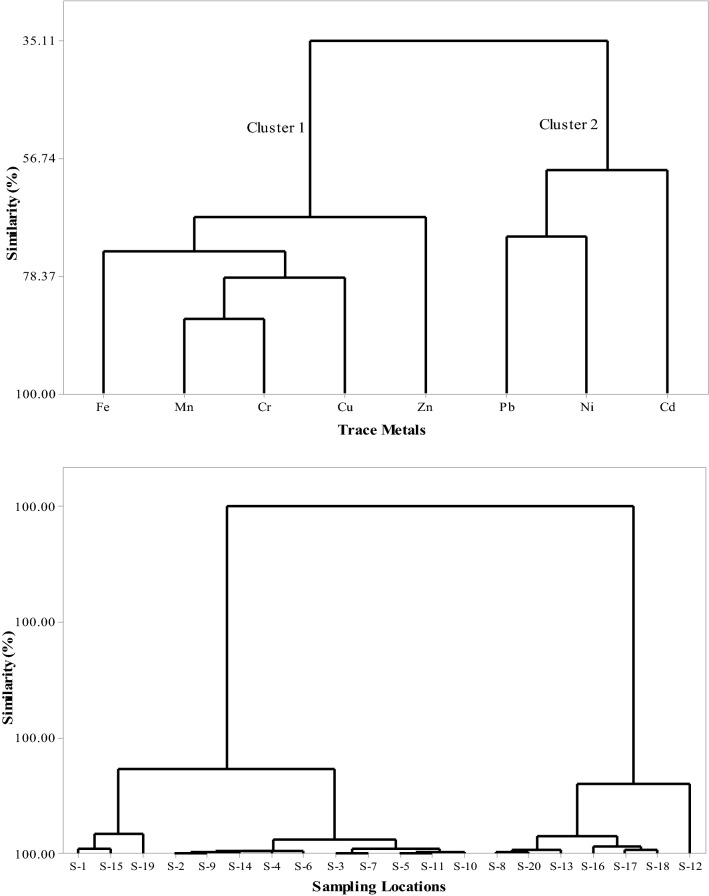
Dendrogram of sampling locations and trace metals.

Hierarchical clustering of the sampling sites and trace metals using complete linkage was constructed to analyze the similarities among the sampling sites and trace metals (Fig. 4). This study also supported the findings of correlations and principal components analysis. The sampling locations were clustered into two groups: group 1 (S-1 to S-11, S-14, S-15, S-19) and group 2 with the rest of the samples. This association emphasized variations in the degree of contamination between Poshur river adjacent mangrove and other Sundarbans areas. Similarly, trace metals were clustered into two clusters based on their enrichment in soils. Lead, cadmium, and nickel were in cluster 2, which are three dominant contaminants in the Sundarbans soils. The multivariate analysis underlined the need for environmental risk assessment of these major contaminants in the Sundarbans soils.

### Risk assessment of trace metals

**Figure 5:**
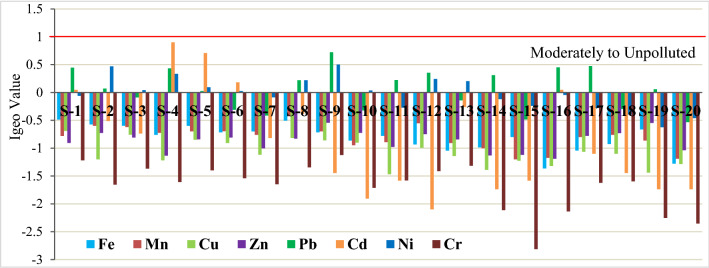
Trace metals geo-accumulation (I-geo) in the Sundarbans soils.

Only total concentrations of soil trace metals cannot depict the extent of contamination and whether the sources of pollutants are natural or anthropogenic^65^. Thus, enrichment factor (EF) and geo-accumulation index (I-geo) were used to estimate the natural or anthropogenic, or mixed sources of heavy metals using Fe as a reference value^41^. Among the eight trace metals, Pb, Cd, and Ni showed a positive value of I-geo (Fig. 5). This result suggested that Sundarbans soils are uncontaminated with Mn, Cu, Zn, and Cr, while moderately contaminated with Pb, Cd, and Ni alone or combinedly. Moderate Pb, Ni, and Cd contaminations have previously been reported in Indian Sundarbans by several researchers^14,21,22^.

**Figure 6:**
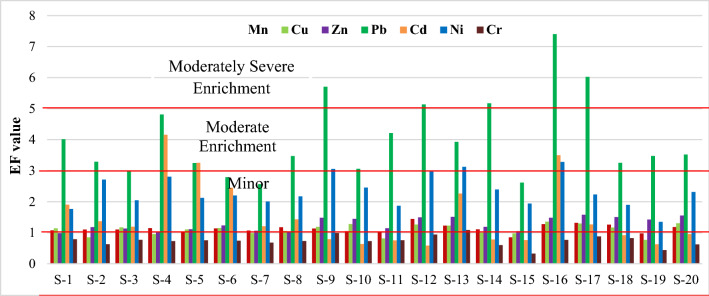
Enrichment of seven trace metals in the Sundarbans soils.

An EF value of < 2 indicates natural origin, whereas a value of > 2 indicates anthropogenic sources^14,41^. Based on the data in Fig. 6, it can be said that Sundarbans soils were in the range of minor enrichments with Mn, Cu, and Zn. The soils were classified as poor to moderate enrichment for Ni and Cd and moderate to severe enrichment for Pb. Hence, Sundarbans soils are in the class of moderate to moderately severe pollution due to anthropogenic sources.

**Figure 7:**
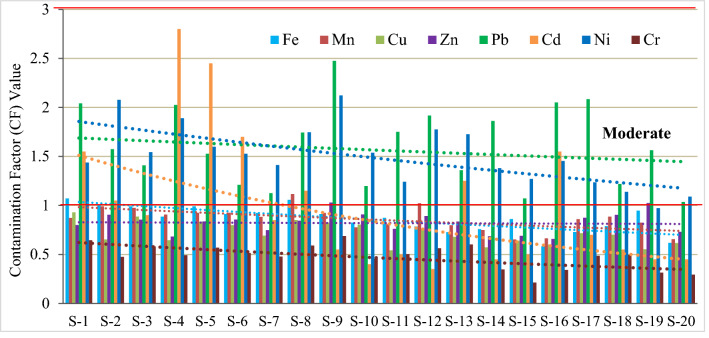
Contamination factor (CF) values of eight trace metals in studied Sundarbans soils.

The CF values for nickel and lead in all the studied mangrove soils, for cadmium in S-1-2, S-4-6, S-8, S-13, S-16, for iron in S-1, S-2, S-3, S-5, S-8, for manganese in S-8, S-12, and Zn in only S-9 was higher than 1 (Fig. 7). These metals possessed a moderate contamination risk in the locations mentioned above according to the classification. In the current study, copper and chromium maintained a low contamination risk (CF < 1) in all the soils. It can be concluded from Fig. 7 that eight studied locations (S-1, S-2, S-4, S-5, S-6, S-8, S-13, S-16) were contaminated by three contaminants, namely Pb, Cd, Ni and the rest locations by only Ni and Pb. All the risk assessment parameters supported the results of each other and therefore, it can be said that the Sundarbans soils were moderately polluted with Pb, Ni, and Cd and unpolluted/low polluted with the rest of the trace metals.

**Figure 8:**
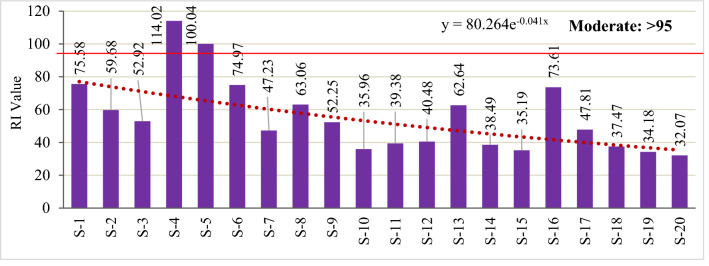
Potential ecological risk (RI) of studied trace metals in the Sundarbans soils.

Quantification of the ecological risk was done using RI after considering trace metals concentrations, toxic response factors, and ecological factors. However, RI results showed that the above contaminants were still within the low potential ecological risk categories in most of the studied sites (RI: 32.07–75.58), although S-4 (RI = 114.04) and S-5 (RI = 100.04) showed a moderate level of environmental risk in the Sundarbans ecosystem (Fig. 8). The exponential curve for all the risk assessment parameters highlighted that contaminations level decreased from S-1 to S-20 possibly due to increasing distance from the point and nonpoint pollutant sources. This study also depicted that the Posur river adjacent area was more contaminated than the other areas of Sundarbans.

**Figure 9:**
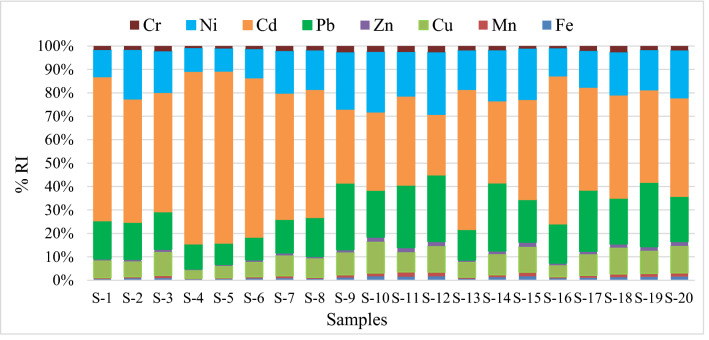
Contribution of individual trace metals to RI in the Sundarbans soils.

The mean role of individual trace metals in RI ranged from 0.5–1.7% Fe, 0.4–2.0% Mn, 3.4–13.4% Cu, 0.7–2.0% Zn, 9.2–29.1% Pb, 25.9–73.7% Cd, 9.6–26.4% Ni and 0.9–2.7% Cr (Fig. 9). It is evident that Cd among the metals posed the highest ecological threats to the Sundarbans soils. Though Cd concentration was lowest compared to the other seven metals, they posed a high risk due to their high toxicity, nonbiodegradability, and long persistence time in the environment. High Cd contamination risks were also reported in several recent risk assessment studies^28,49^.

The risk assessment indices showed that the higher concentrations of these toxic metals were probably driven by transportation phenomena, shipbreaking activities, smelting factories, and untreated industrial waste discharges in nearby areas of Sundarbans mangrove in Bangladesh.

## Conclusion

Sundarbans soils are in the high-risk category of trace metals pollution, as soils have high retention capacity due to their finer particle size and high organic carbon content. The combined use of I-geo, EF, CF, CD, and RI indicated that the studied locations were uncontaminated to poorly contaminated with Zn, Cu, Cr, Fe, and Mn and moderately contaminated with either Pb, Cd, and Ni or only Pb and Ni. Moreover, S-4 and S-5 were the most contaminated among the twenty locations but still had a moderate toxicity index. The sequence of examined trace metals concentrations was Fe > Mn > Ni > Zn > Cr > Cu > Pb > Cd while, pollution sequence was Pb > Ni > Cd > Fe > Mn > Zn > Cu and contamination extent decreased from upstream to downstream. The Correlations, principal component analysis (PCA) and hierarchical cluster analysis (HCA) quantified the relationship among trace metals and their possible origins. The PCA and HCA analyses separated Pb, Cd, and Ni from other metals and grouped them into a distinct cluster. Anthropogenic stresses from urbanization, industrialization, navigations, shipbreaking, metallurgical industries were mainly responsible for the pollution. In addition, some other trace metals may also release from these sources and are expected to worsen the pollution level since metals are not unique to a certain source. Future studies should focus on continuous monitoring of toxic trace metals pollution particularly Pb, Cd, and Ni and emphasis on the adoption of appropriate remediation strategies to reduce the concentrations into a target value. This study can be used as baseline data for future monitoring and conservation in the Sundarbans mangrove forest.

## Data Availability

The datasets used and/or analyzed during the current study are available from the corresponding author on reasonable request.
